# Editorial: Neglected and underutilized crop species for sustainable food and nutritional security: prospects and hidden potential

**DOI:** 10.3389/fpls.2023.1358220

**Published:** 2024-01-09

**Authors:** Omena Bernard Ojuederie, David Okeh Igwe, Ndiko Ndomele Ludidi, Beckley Ikhajiagbe

**Affiliations:** ^1^ Department of Biological Sciences, Biotechnology Unit, Faculty of Science, Kings University, Ode-Omu, Osun, Nigeria; ^2^ Food Security and Safety Focus, Faculty of Natural and Agricultural Sciences, North-West University, Mmabatho, South Africa; ^3^ Plant Pathology and Plant-Microbe Biology Section, School of Integrative Plant Science, Cornell University, Ithaca, NY, United States; ^4^ Department of Biotechnology, University of the Western Cape, Bellville, South Africa; ^5^ Department of Plant Biology and Biotechnology, Faculty of Science, University of Benin, Benin City, Edo, Nigeria

**Keywords:** food security, genetic improvement, orphan crops, sustainable agriculture, zero hunger

The exploration of neglected and underutilized crop species (NUCs) is indeed crucial in tackling global food insecurity. These nutrient-rich, climate-resilient crops, often disregarded for limited commercial value, hold the key to combating malnutrition and boosting food security, especially in vulnerable regions. These crops, which have not been previously categorized as major crops and are mainly confined to smallholder farming areas, are nutrient-dense, climate-resilient, and locally adaptable (Li and Siddique, 2020; Mudau et al., 2022). The erosion of these crops can hinder the nutritional status and food security of the poor, and their greater use can augment nutrition and fight hidden hunger (Dansi et al., 2012; Ojuederie et al., 2015; Joy and Siddhuraju, 2017). It is crucial that we recognize the hidden potential of these crops and harness them to achieve a more sustainable future.

This editorial spotlight promising research showcasing the hidden potential of NUCs and exploring their utilization through modern advancements. The range of research showcased in this editorial on the Research topic *‘Neglected and Underutilized Crop Species for Sustainable Food and Nutritional Security: Prospects and Hidden Potential’* is impressive and covers various aspects of these crops, from their genetic improvement to their potential applications in diverse fields. The Research Topic consists of 9 publications: 6 original research articles and 3 reviews focusing on the genetic improvement, conservation and utilization of some NUCs in addressing global food and nutrition challenges.

*Citrus grandis* (L.) Osbeck, commonly called pomelo, is an underutilized citrus fruit, whose potential as a source of flavonoids, phenols, and antioxidants, have been overlooked. The investigation into drying methods for *Citrus grandis* fruits by Kumar et al. sheds light on optimizing processes to retain their bioactive compounds, potentially opening avenues for functional food development and nutraceuticals. In the parched lands where water is a precious commodity, foxtail millet stands tall, a beacon of hope for farmers and food security.

The research by Singh et al. investigated the potential for improving foxtail millet (*Setaria italica* L. beauv) yields in rainfed conditions through germplasm assessment and analysis. From this research, five superior genotypes emerged through genetic analysis: Kangni-7, Kangni-1, Kangni-6, Kangni-5, and Kangni-4 offering a promising approach to revolutionize its production under harsh conditions and bolster food security and enhance livelihoods.

The effectiveness of using mock genomes for conducting genomic studies in orphan crops, which lack a reference genome was investigated by Machado et al. The genotyping-by-sequencing-Mock approach provided comparable results to standard methods in the areas of genetic diversity studies, dividing heterotic groups, selecting testers, and predicting the performance of single crosses. The study offers a promising approach for leveraging genomic technologies to improve the development of orphan crops, which can have significant benefits for global food security and nutrition. Duckweed, with rapid growth and high protein content, shows potential for biofuel production, animal feed, and wastewater treatment.

The assessment of genetic variability among duckweed clones using DNA barcoding markers has been a subject of interest (Borisjuk et al., 2014; Al-Dakhil et al., 2021). Exploring DNA barcoding techniques and assessing the biomass accumulation rates of duckweed species, particularly of native Iranian types, offer promising opportunities for biotechnological applications.

Taghipour et al. identified four duckweed Iranian species using two standard chloroplast markers and assessed their growth rates, emphasizing their potential for sustainable biotechnological solutions. Growth rates of selected duckweed clones were found to be higher than common crops, demonstrating their potential for large-scale biomass production. As a result of the high protein content of the Native Iranian duckweed, it can be utilized as a sustainable source of protein for food and feed applications and in the development of valuable recombinant proteins.

The genetic dissection of agronomic traits in Andean lupin (*L. mutabilis*) and Bambara groundnut, offer invaluable insights for breeding improved varieties and promoting these crops’ consumption. We highlight the potential impact of the research of Gulisano et al. on Andean lupin improvement for sustainable agriculture. Their research identifies genetic factors for breeding adapted Andean lupin varieties, laying the groundwork for its successful cultivation in Europe and establish a foundation for further research on plant development and phenology in *L. mutabilis*, paving the way for improved breeding programs and a better understanding of plant development in this species.

Understanding the genetic basis of drought tolerance in Bambara groundnut is crucial for breeding programs. Odesola et al. considered both genetic diversity and environmental interactions for effective breeding programs. The study paves the way for developing improved Bambara varieties resilient to climate change through targeted breeding approaches.

Underutilized legumes (NULs) in Africa are valuable sources of nutrition and bioactive compounds offering health-promoting benefits. Despite their nutritional value, they remain neglected and underutilized. Legume seeds possessing bioactive compounds with antioxidant activity could lessen the negative impact of oxidative stress, and enhance the well-being of man. Popoola et al. reviewed selected NULs and their nutritional, functional, and bioactive properties. their potential and challenges, proposing strategies for increased utilization and their role in developing sustainable and healthy food systems and strategies proposed for their increased exploitation.

Moth bean, a highly adaptable and nutritious crop, is highlighted by Kanishka et al. as a climate-smart crop for food security and income generation. This underscores the importance of further research and development efforts. The authors advocates for increased research and development to unlock its full potential, leveraging its unique strengths and untapped potential to address agricultural challenges in a changing climate.

The review of Kudapa et al. focusing on biofortification of millets through genetic and genomic interventions showcases the potential of cutting-edge technologies like CRISPR-Cas9 in addressing micronutrient malnutrition, presenting a viable solution for sustainable food and nutritional security. Combining genetic and genomic interventions with harnessing the key characteristics of millets, offer promising solutions to combat micronutrient malnutrition and contribute to sustainable food and nutritional security. Overall, this comprehensive range of research efforts highlights the multifaceted approach needed to unlock the hidden potential of neglected and underutilized crops, paving the way for a more sustainable and food-secure future.

## Author contributions

OO: Conceptualization, Writing – original draft, Writing – review & editing. DI: Writing – original draft, Writing – review & editing. NL: Writing – original draft, Writing – review & editing. BI: Writing – original draft, Writing – review & editing.
